# International Committee on Systematics of Prokaryotes Subcommittee on the Taxonomy of Halobacteria and Subcommittee on the Taxonomy of Halomonadaceae: minutes of the joint open meeting, 25 November 2025, Oaxaca, Mexico

**DOI:** 10.1099/ijsem.0.007065

**Published:** 2026-02-06

**Authors:** Aharon Oren, David R. Arahal, Antonio Ventosa

**Affiliations:** 1 Department of Plant and Environmental Sciences, The Institute of Life Sciences, The Hebrew University of Jerusalem, Edmond J. Safra Campus, Jerusalem 9190401, Israel; 2 Department of Microbiology and Ecology, Universitat de València, Valencia 46100, Spain; 3 Department of Microbiology and Parasitology, Universidad de Sevilla, Sevilla 41012, Spain

**Keywords:** *Halobacteria*, *Halomonadaceae*, International Committee on Systematics of Prokaryotes (ICSP) Subcommittee, minutes

## Minute 1: call to order

The meeting was held at the Hotel Fortín Plaza, Oaxaca, Mexico. The chair, A. Ventosa, opened the meeting at 14.45.

## Minute 2: record of attendance

The subcommittee members present were A. Ventosa (Spain, Chair, Subcommittee on the Taxonomy of *Halobacteria* and Subcommittee on the Taxonomy of *Halomonadaceae*), A. Oren (Israel, Secretary, Subcommittee on the Taxonomy of *Halobacteria*), R.R. de la Haba, C. Sánchez-Porro (Spain) and R.H. Vreeland (USA). Apologies were received from D.R. Arahal (Spain, Secretary, Subcommittee on the Taxonomy of *Halomonadaceae*), H.-L. Cui (PR China, Vice-Chair, Subcommittee on the Taxonomy of *Halobacteria*), R.R. Colwell (USA), M.L. Dyall-Smith (Australia), R. Montalvo-Rodriguez (Puerto Rico), M.R. Mormile (USA), H. Stan-Lotter (Austria) and B.J. Tindall (Germany). The meeting was further attended by A.Y. Miranda Jimenez (Bolivia), J. Jehlička (Czech Republic), Y. Fernández, M.I. Infante, A. Vargas Fernández (Dominican Republic), L.A. Yarzábal Rodríguez (Ecuador), V. Alcopor Roman, I. Arroyo-Herrera, R.M. Camacho Ruíz, M. Camarena Flores, A. [Abigail] Coria Guadarrama, A. [Atalia] Coria Guadarrama, C. Gómez Urzúa, D.N. Cruz Luna, R. Escalera Pastor, A.J. Gómez Sánchez, J. Guevara Luna, G.V. Guizar Sánchez, I.A. Gutiérrez Reyna, A. de J. Hernández Gómez, L.M. Hernández-Soto, M.A. Jacinto-Sosa, M.A. Lago Lestón, C. López Sánchez, P. Magaña Mejía, A. Méndez Zamora, A. Miranda Ruiz, M.G. Encarnacion Molina, K. Muñoz, S. Ortiz Sotelo, E.T. Quintana, N. Ramírez Durán, H. Ramírez Saad, Y.V. Rodríguez, M. del R. Sánchez Carbente, H. Silva Jiménez, M.N. Silvar Ortiz, M.S. Vásquez Murrieta (Mexico), J. Hamm (The Netherlands), R.A. Batista García, M.P. Corral Villa, M. Carillo-Bautista, J.M. Martinez Lozano, F. Santos and T. Viver (Spain).

## Minute 3: appointment of a secretary

A. Oren (Secretary, Subcommittee on the Taxonomy of *Halobacteria*) was appointed secretary of the joint subcommittee meeting.

## Minute 4: approval of agenda

The agenda of the meeting was approved.

## Minute 5: minutes of previous meetings

The minutes of the joint open meeting of the International Committee on Systematics of Prokaryotes (ICSP) Subcommittee on the Taxonomy of *Halobacteria* and Subcommittee on the Taxonomy of *Halomonadaceae*, held on 28 June 2022 in Alicante, Spain, were published (Oren A, Arahal DR, Ventosa A, *Int J Syst Evol Microbiol* 2022;72:005584). On 12 May 2022, the ICSP Subcommittee on the Taxonomy of *Halobacteria* held a closed meeting via Zoom (Oren A, Ventosa A. *Int J Syst Evol Microbiol* 2022;72:005499).

## Minute 6: in memoriam Larry Hochstein

Lawrence I. (Larry) Hochstein, a long-time member of the *Halobacteriaceae* subcommittee, passed away in July 2024 at the age of 96. The species *Halorubrum hochsteinianum* was named after him (Vreeland RH, Sun Y-P, Wang B-B, Hou J, Cui H.-L. *Extremophiles* 2024;28:1). An obituary for Larry has been published (Vreeland RH, Oren A, Ventosa A. *Extremophiles* 2025;29:5). R.H. Vreeland spoke in memory of Larry.

## Minute 7: report of the chair

A. Ventosa explained the work of the ICSP as one of the ComCofs (Committees, Commissions and Federations) of the International Union of Microbiological Societies (IUMS), its Bacteriology and Applied Microbiology (BAM) Division and the functions of the two subcommittees on taxonomy that deal with halophilic prokaryotes.

## Minute 8: new taxa within the class *Halobacteria*

In view of the large number of new species published in the past years, no full list, similar to those presented at previous joint open subcommittees meetings, was presented. Full information can be retrieved from the List of Prokaryotic Names with Standing in Nomenclature (LPSN; https://www.lpsn.dsmz.de). The curator of LPSN has prepared annual updates of the lists of newly published names relevant to the subcommittees. Instead, we have here listed the names of genera and higher taxa published between June 2022 and October 2025, as well as reclassifications, emendations and synonymy proposals. The list of orders, families and genera given in Table 1 of the 2024 Proposed Minimal Standards document (Cui *et al*., *Int J Syst Evol Microbiol* 2024;74:006290) still provides an excellent overview, and the taxa marked with an asterisk below should be added to update those lists.

The parent taxon of the class *Halobacteria* is the phylum *Halobacteriota* Chuvochina 2024 (Validation List 215; effective publication: Chuvochina *et al*., *FEMS Microbiol Lett* 2023;370:fnad071). The type genus is *Halobacterium* Elazari-Volcani 1957 (Approved Lists 1980).

According to the LPSN, the phylum name *Halobacteriota* is a synonym of *Methanobacteriota* Garrity and Holt 2023. The phylum is classified in the kingdom *Methanobacteriati* (Garrity and Holt 2023) Oren and Göker 2024.

One new order was added to the class *Halobacteria*:

*- Halorutilales* Durán-Viseras *et al*. 2023 (Validation List 213; effective publication: Durán-Viseras *et al*., *mSystems* 2023;8:01198-22). The type genus is *Halorutilus* Durán-Viseras *et al*. 2023.

Four new families were proposed in the order *Halobacteriales*:

- *Natronoarchaeaceae* Sorokin *et al*. 2023 (Validation List 211; effective publication: Sorokin *et al., Syst Appl Microbiol* 2022;45:126356). The type genus is *Natronoarchaeum* Shimane *et al*. 2010.
- *Haladaptataceae* Cui *et al*. 2023 (*Int J Syst Evol Microbiol* 2023;73:5984). The type genus is *Haladaptatus* Savage *et al*. 2007. *Haladaptataceae* Chuvochina *et al*. 2024 (Validation List 215; effective publication: Chuvochina *et al*., *FEMS Microbiol Lett* 2023;370:fadn071) is a later homonym and is illegitimate. The genera *Haladaptatus*, *Halomicrococcus* and *Halorussus*, earlier classified in the *Halobacteriaceae*, were transferred to this new family.
- *Halalkalicoccaceae Mao *et al*. 2025 (Validation List 223; effective publication: Mao *et al*., *Syst Appl Microbiol* 2025;48:126577). The type genus is *Halalkalicoccus* Xue *et al*. 2005. The genus *Halalkalicoccus*, earlier classified in the *Halococcaceae*, was transferred to this new family.
- *Natronomonadaceae Zhu *et al*. 2025 (Validation List 224; effective publication: Zhu *et al*., *Syst Appl Microbiol* 2025;48:126592). The type genus is *Natronomonas* Kamekura *et al*. 1997. The genera *Halocatena*, *Haloglomus*, *Halomarina*, *Halorarius*, *Halosegnis*, *Natronomonas* and *Salinirubellus*, earlier classified in the *Haloarculaceae*, were transferred to this new family.

One new family was proposed in the new order *Halorutilales*:

*- Halorutilaceae* Durán-Viseras *et al*. 2023 (Validation List 213; effective publication: Durán-Viseras *et al*., *mSystems* 2023;8:01198-22). The type genus is *Halorutilus* Durán-Viseras *et al*. 2023.

The following new genera were described:

In the family *Halalkalicoccaceae*:

- *Natronorarus Mao *et al*. 2025 (Validation List 223; effective publication: Mao *et al*., *Syst Appl Microbiol* 2025;48:126577). The type species is *Natronorarus salvus* Mao *et al*. 2025. Recommended three-letter abbreviation: *Nrs*.

In the family *Haloarculaceae*:

- *Halorarius* Sun *et al*. 2023 (Validation List 211; effective publication: Sun *et al*., *Front Mar Sci* 2023;10:1105929). The type species is *Halorarius litoreus* Sun *et al*. 2023. Recommended three-letter abbreviation: *Hor*.
- *Salella* Deshmukh and Oren 2023 (*Int J Syst Evol Microbiol* 2023;73:5901). Replacement name for the illegitimate name *Sala* Song *et al*. 2023 (Validation List 209; effective publication: Song *et al., J Microbiol* 2022;60:899–904). The type species is *Salella cibi* (Song *et al*. 2023) Deshmukh and Oren 2023. Recommended three-letter abbreviation: *Sll*.

In the family *Halobacteriaceae*:

- *Actinarchaeum corrig. Tang *et al*. 2024 (Validation List 215; effective publication: Tang *et al*., *Nat Commun* 2023;14:1827). The type species is *Actinarchaeum halophilum* corrig. Tang *et al*. 2024. Recommended three-letter abbreviation: *Aah*.
- *Halospeciosus Li *et al*. 2024 (*Int J Syst Evol Microbiol* 2024;74:006620). The type species is *Halospeciosus flavus* Li *et al*. 2024. Recommended three-letter abbreviation: *Hsc*.

In the family *Haloferacaceae*:

- *Halalkalirubrum* Zuo *et al*. 2022 (Validation List 207; effective publication: Zuo *et al*., *Antonie van Leeuwenhoek* 2021;114:83–94). The type species is *Halalkalirubrum salinum* Zuo *et al*. 2022. Recommended three-letter abbreviation: *Hak*.
- *Natronocalculus* Sorokin *et al*. 2022 (Validation List 211; effective publication: Sorokin *et al*., *Syst Appl Microbiol* 2022;45:126336). The type species is *Natronocalculus amylovorans* Sorokin *et al*. 2023. Recommended three-letter abbreviation: *Ncl*.
- *Halorarum Tan *et al*. 2024 (Validation List 218; effective publication: Tan *et al*., *Extremophiles* 2024;28:22). The type species is *Halorarum halophilum* (Cui *et al*. 2021) Tan *et al*. 2024. Recommended three-letter abbreviation: *Hrm*.
- *Salinirarus* Zhang *et al*. 2025 (Validation List 222; effective publication: Zhang *et al*., *FEMS Microbiol Lett* 2024;371:fnae075). The type species is *Salinirarus marinus* Zhang *et al.* 2025. Recommended three-letter abbreviation: *Sra*.

In the family *Natrialbaceae*:

- *Halosolutus* Sun *et al*. 2022 (*Int J Syst Evol Microbiol* 2022;72:005598). The type species is *Halosolutus amylolyticus* Sun *et al*. 2022. Recommended three-letter abbreviation: *Hss*.
- *Natronosalvus* Tan *et al*. 2023 (*Int J Syst Evol Microbiol* 2023;73:006036). The type species is *Natronosalvus halobius* Tan *et al*. 2023. Recommended three-letter abbreviation: *Nsv*.
- *Natronobeatus* Li *et al*. 2023 (Validation List 214; effective publication: Li *et al*., *Extremophiles* 2023;27:15). The type species is *Natronobeatus ordinarius* Li *et al*. 2023. Recommended three-letter abbreviation: *Nbs*.
- *Natrononativus* Li *et al*. 2023 (Validation List 214; effective publication: Li *et al*., *Extremophiles* 2023;27:15). The type species is *Natrononativus amylolyticus* Li *et al*. 2023. Recommended three-letter abbreviation: *Nbs*.
- *Salinilacihabitans* Li *et al*. 2023 (Validation List 214; effective publication: Li *et al*., *Extremophiles* 2023;27:15). The type species is *Salinilacihabitans rarus* Li *et al*. 2023. Recommended three-letter abbreviation: *Slc*.
- *Halomontanus* Mao *et al*. 2024 (Validation List 218; effective publication: *Syst Appl Microbiol* 2024;47:126500). The type species is *Halomontanus rarus* Mao *et al*. 2024. Recommended three-letter abbreviation: *Hmt*.
- *Natronoglomus Sorokin *et al*. 2025 (Validation List 221; effective publication: *Front Microbiol* 2024;15:1364606). The type species is *Natronoglomus mannanivorans* Sorokin *et al*. 2025. Recommended three-letter abbreviation: *Ngm*.
- *Halovalidus Dong *et al*. 2025 (Validation List 222; effective publication: *Extremophiles* 2024;28:47). The type species is *Halovalidus salilacus* Dong *et al*. 2025. Recommended three-letter abbreviation: *Hvd*.

In the family *Natronoarchaeaceae*:

- *Natranaeroarchaeum* Sorokin *et al*. 2023 (Validation List 211; effective publication: *Syst Appl Microbiol* 2022;45:126356). The type species is *Natranaeroarchaeum sulfidigenes* Sorokin *et al*. 2023. Recommended three-letter abbreviation: *Naa*.

In the family *Halorutilaceae*:

*- Halorutilus* Durán-Viseras *et al*. 2023 (Validation List 213; effective publication: Durán-Viseras *et al*., *Int J Syst Evol Microbiol* 2023;73:5997). The type species is *Halorutilus salinus* Durán-Viseras *et al*. 2023. Recommended three-letter abbreviation: *Hrt*.

Reclassifications and changes in taxonomic opinion:

- *Halobellus ramosii* Pérez-Davó *et al*. 2015 was proposed as a heterotypic synonym of *Halobellus inordinatus* Qiu *et al*. 2013 (Quadri *et al*., *Curr Microbiol* 2024;81:216).
- *Haloferax alexandrinum* corrig. Askar and Ohta 2002 was proposed as a heterotypic synonym of *Haloferax volcanii* (Mullakhanbhai and Larsen 1975) Torreblanca *et al*. 1986 (Quadri *et al*., *Curr Microbiol* 2024;81:216).
- *Haloferax lucentense* corrig. Gutierrez *et al*. 2004 was proposed as a heterotypic synonym of *Haloferax volcanii* (Mullakhanbhai and Larsen 1975) Torreblanca *et al*. 1986 (Quadri *et al*., *Curr Microbiol* 2024;81:216).
- *Halomicroarcula onubensis* Straková *et al*. 2024 was reclassified as *Haloarcula onubensis* comb. nov. (Straková *et al*., *Int J Syst Evol Microbiol* 2024;74:006510).
- *Halomicroarcula saliterrae* Straková *et al*. 2024 was reclassified as *Haloarcula saliterrae* comb. nov. (Straková *et al*., *Int J Syst Evol Microbiol* 2024;74:006510).
- *Salella cibi* Deshmukh and Oren 2023 (*Int J Syst Evol Microbiol* 2023;73:5901) was proposed as a replacement name for the illegitimate name *Salella cibi* Song *et al*. 2023 (Validation List 209; effective publication: Song *et al. J Microbiol* 2022;60:899–904).

## Minute 9: new taxa of Nanohaloarchaeota

Since the subcommittee meeting in Alicante in 2022, the following names of new taxa of Nanohaloarchaeota were published:

Class:

- ‘*Candidatus* Nanohaloarchaeia’ – a name mentioned incidentally by La Cono *et al*., *Microbial Biotechnol* 2023;16:1803–1822. Type genus: ‘*Candidatus* Nanohalarchaeum’.
- La Cono *et al*. (*Microbial Biotechnol* 2023;16:1803–1822) classified the *Candidatus* genera Nanohalovita, Nanopetraeus, Nanohalobium and Nanosalina in the ‘*Candidatus* Nanohalobiia’ class.

Family:

- ‘*Candidatus* Nanoanaerosalinaceae’ Zhao *et al*. 2022 (*mSystems* 2022;7:00669-22). Type genus: ‘*Candidatus* Nanoanaerosalina’.
- ‘*Candidatus* Nanohalalkaliarchaeceae’ Zhao *et al*. 2022 (*mSystems* 2022;7:00669-22). Type genus: ‘*Candidatus* Haloalkaliarchaeum’.

Genus:

- ‘*Candidatus* Nanoanaerosalina’ Zhao *et al*. 2022 (*mSystems* 2022;7:00669-22). Type species: ‘*Candidatus* Nanoanaerosalina halalkaliphila’.
- ‘*Candidatus* Nanohalalkaliarchaeum’ Zhao *et al*. 2022 (*mSystems* 2022;7:00669-22). Type species: ‘*Candidatus* Nanohalalkaliarchaeum halalkaliphilum’.
- ‘*Candidatus* Nanohalovita’ Reva *et al*. 2023 (*Front Microbiol* 2023;14:1182464). Type species: ‘*Candidatus* Nanohalovita haloferacivicina’.
- ‘*Candidatus* Nanohalococcus’ Reva *et al.* 2023 (*Front Microbiol* 2023;14:1182464). Type species: ‘*Candidatus* Nanohalococcus occultus’.

Species:

- ‘*Candidatus* Nanoanaerosalina halalkaliphila’ Zhao *et al*. 2022 (*mSystems* 2022;7:00669-22).
- ‘*Candidatus* Nanohalalkaliarchaeum halalkaliphilum’ Zhao *et al*. 2022 (*mSystems* 2022;7:00669-22).
- ‘*Candidatus* Nanohalovita haloferacivicina’ Reva *et al*. 2023 (*Front Microbiol* 2023;14:1182464).
- ‘*Candidatus* Nanohalococcus occultus’ Reva *et al*. 2023 (*Front Microbiol* 2023;14:1182464).

## Minute 10: new taxa within the family *Halomonadaceae*

The following names of new genera of *Halomonadaceae* were published since the Alicante 2022 subcommittee meeting:

- *Billgrantia* de la Haba *et al*. 2024 (Validation List 216; effective publication: de la Haba *et al*., *Front Microbiol* 2023;14:1293707). The type species is *Billgrantia desiderata* (Berendes *et al*. 1997) de la Haba *et al*. 2024.
- *Bisbaumannia* de la Haba *et al*. 2024 (Validation List 216; effective publication: de la Haba *et al*., *Front Microbiol* 2023;14:1293707). The type species is *Bisbaumannia pacifica* (Baumann *et al*. 1972) de la Haba *et al*. 2024.
- *Franzmannia* de la Haba *et al*. 2024 (Validation List 216; effective publication: de la Haba *et al*., *Front Microbiol* 2023;14:1293707). The type species is *Franzmannia pantelleriensis* (Romano *et al*. 1997) de la Haba *et al*. 2024.
- *Litchfieldella* de la Haba *et al*. 2024 (Validation List 216; effective publication: de la Haba *et al*., *Front Microbiol* 2023;14:1293707). The type species is *Litchfieldella anticariensis* (Martínez-Cánovas *et al*. 2004) de la Haba *et al*. 2024.
- *Onishia* de la Haba *et al*. 2024 (Validation List 216; effective publication: de la Haba *et al*., *Front Microbiol* 2023;14:1293707). The type species is *Onishia taeanensis* (Lee *et al*. 2005) de la Haba *et al*. 2024.
- *Vreelandella* de la Haba *et al*. 2024 (Validation List 216; effective publication: de la Haba *et al*., *Front Microbiol* 2023;14:1293707). The type species is *Vreelandella aquamarina* (ZoBell and Upham 1944) de la Haba *et al*. 2024.

Reclassifications and changes in taxonomic opinion:

- *Chromohalobacter salexigens* Arahal *et al*. 2001 was proposed as a heterotypic synonym of *Chromohalobacter israelensis* (Huval *et al*. 1996) Arahal *et al*. 2001 (de la Haba *et al., Front Microbiol* 2023;14:1293707).
- *Cobetia amphilecti* Romanenko *et al*. 2013 emend. Nedashkovskaya *et al*. 2024 (*Biomolecules* 2024;14:196).
- *Cobetia litoralis* Romanenko *et al*. 2013 was proposed as a heterotypic synonym of *Cobetia amphilecti* Romanenko *et al.* 2013 (Nedashkovskaya *et al*. 2024 *Biomolecules* 2024;14:196).
- *Cobetia marina* (Cobet *et al*. 1970) Arahal *et al*. 2002 emend. Nedashkovskaya *et al*. 2024 (*Biomolecules* 2024;14:196).
- *Cobetia pacifica* Romanenko *et al*. 2013 was proposed as a heterotypic synonym of *Cobetia marina* (Cobet *et al*. 1970) Arahal *et al*. 2002 (Nedashkovskaya *et al*. 2024 *Biomolecules* 2024;14:196).
- *Halomonadaceae* Franzmann *et al*. 1989 emend. Flores-Félix *et al*. 2025 (*Mol Phylogenet Evol* 2025;206:108321). According to this emendation, the family belongs to the order *Pseudomonadales*, and it comprises the genera *Allopseudospirillum*, *Carnimonas*, *Chromohalobacter*, *Cobetia*, *Halomonas*, *Halotalea*, *Kushneria*, *Larsenimonas*, *Marinospirillum*, *Modicisalibacter*, *Pistricoccus*, *Salinicola*, *Terasakiispira* and *Zymobacter*.
- *Halomonas alkaliantarctica* Poli *et al*. 2011 was proposed as a heterotypic synonym of *Halomonas neptunia* Kaye *et al*. 2004 (de la Haba *et al., Front Microbiol* 2023;14:1293707).
- *Halomonas alkaliphila* Romano *et al*. 2007 was reclassified as *Vreelandella alkaliphila* comb. nov. (Validation List 216; effective publication: de la Haba *et al*., *Front Microbiol* 2023;14:1293707).
- *Halomonas andesensis* Guzmán *et al*. 2010 was reclassified as *Vreelandella andesensis* comb. nov. (Validation List 216; effective publication: de la Haba *et al*., *Front Microbiol* 2023;14:1293707).
- *Halomonas anticariensis* Martínez-Cánovas *et al*. 2004 was reclassified as *Litchfieldella anticariensis* comb. nov. (Validation List 216; effective publication: de la Haba *et al*., *Front Microbiol* 2023;14:1293707).
- *Halomonas antri* So *et al*. 2022 was reclassified as *Billgrantia antri* comb. nov. (Validation List 216; effective publication: de la Haba *et al*., *Front Microbiol* 2023;14:1293707).
- *Halomonas aquamarina* (ZoBell and Upham 1944) Dobson and Franzmann 1996 was reclassified as *Vreelandella aquamarina* comb. nov. (Validation List 216; effective publication: de la Haba *et al*., *Front Microbiol* 2023;14:1293707).
- *Halomonas arcis* Xu *et al*. 2007 was reclassified as *Vreelandella arcis* comb. nov. (Validation List 216; effective publication: de la Haba *et al*., *Front Microbiol* 2023;14:1293707).
- *Halomonas axialensis* Kaye *et al*. 2004 was proposed as a heterotypic synonym of *Halomonas aquamarina* (ZoBell and Upham 1944) Dobson and Franzmann 1996 (de la Haba *et al., Front Microbiol* 2023;14:1293707).
- *Halomonas azerbaijanica* Kazemi *et al*. 2021 was reclassified as *Billgrantia azerbaijanica* comb. nov. (Validation List 216; effective publication: de la Haba *et al*., *Front Microbiol* 2023;14:1293707).
- *Halomonas azerica* Wenting *et al*. 2021 was reclassified as *Vreelandella azerica* comb. nov. (Validation List 216; effective publication: de la Haba *et al*., *Front Microbiol* 2023;14:1293707).
- *Halomonas bachuensis* Xiao *et al*. 2021 was reclassified as *Billgrantia bachuensis* comb. nov. (Validation List 216; effective publication: de la Haba *et al*., *Front Microbiol* 2023;14:1293707).
- *Halomonas boliviensis* Quillaguamán *et al*. 2004 was reclassified as *Vreelandella boliviensis* comb. nov. (Validation List 216; effective publication: de la Haba *et al*., *Front Microbiol* 2023;14:1293707).
- *Halomonas campisalis* Mormile *et al*. 2000 was reclassified as *Billgrantia campisalis* comb. nov. (Validation List 216; effective publication: de la Haba *et al*., *Front Microbiol* 2023;14:1293707).
- *Halomonas daqingensis* Wu *et al*. 2008 was proposed as a heterotypic synonym of *Halomonas desiderata* Berendes *et al*. 1997 (Shang *et al*., *Syst Appl Microbiol* 2022;45:126351).
- *Halomonas desiderata* Berendes *et al*. 1997 was reclassified as *Billgrantia desiderata* comb. nov. (Validation List 216; effective publication: de la Haba *et al*., *Front Microbiol* 2023;14:1293707).
- *Halomonas diversa* Wang *et al*. 2021 was reclassified as *Billgrantia diversa* comb. nov. (Validation List 216; effective publication: de la Haba *et al*., *Front Microbiol* 2023;14:1293707).
- *Halomonas endophytica* Chen *et al*. 2018 was reclassified as *Billgrantia endophytica* comb. nov. (Validation List 216; effective publication: de la Haba *et al*., *Front Microbiol* 2023;14:1293707).
- *Halomonas gomseomensis* Kim *et al*. 2007 was reclassified as *Vreelandella gomseomensis* comb. nov. (Validation List 216; effective publication: de la Haba *et al*., *Front Microbiol* 2023;14:1293707).
- *Halomonas gudaonensis* Wang *et al*. 2007 was reclassified as *Billgrantia gudaonensis* comb. nov. (Validation List 216; effective publication: de la Haba *et al*., *Front Microbiol* 2023;14:1293707).
- *Halomonas hamiltonii* Kim *et al*. 2010 was reclassified as *Vreelandella hamiltonii* comb. nov. (Validation List 216; effective publication: de la Haba *et al*., *Front Microbiol* 2023;14:1293707).
- *Halomonas hydrothermalis* Kaye *et al*. 2004 was proposed as a heterotypic synonym of *Halomonas venusta* (Baumann *et al*. 1972) Dobson and Franzmann 1996 (de la Haba *et al. Front Microbiol* 2023;14:1293707).
- *Halomonas ilicicola* Arenas *et al*. 2009 was reclassified as *Modicisalibacter ilicicola* comb. nov. (Validation List 216; effective publication: de la Haba *et al*., *Front Microbiol* 2023;14:1293707).
- *Halomonas janggokensis* Kim *et al*. 2007 was reclassified as *Vreelandella janggokensis* comb. nov. (Validation List 216; effective publication: de la Haba *et al*., *Front Microbiol* 2023;14:1293707).
- *Halomonas jeotgali* Kim *et al*. 2011 was reclassified as *Vreelandella jeotgali* comb. nov. (Validation List 216; effective publication: de la Haba *et al*., *Front Microbiol* 2023;14:1293707).
- *Halomonas johnsoniae* Kim *et al*. 2010 was proposed as a heterotypic synonym of *Halomonas hamiltonii* Kim *et al*. 2010 (de la Haba *et al., Front Microbiol* 2023;14:1293707).
- *Halomonas kenyensis* Boltyanskaya *et al*. 2008 was reclassified as *Billgrantia kenyensis* comb. nov. (Validation List 216; effective publication: de la Haba *et al*., *Front Microbiol* 2023;14:1293707).
- *Halomonas lactosivorans* Ming *et al*. 2020 was reclassified as *Billgrantia lactosivorans* comb. nov. (Validation List 216; effective publication: de la Haba *et al*., *Front Microbiol* 2023;14:1293707).
- *Halomonas lutea* Wang *et al*. 2008 was reclassified as *Modicisalibacter luteus* comb. nov. (Validation List 216; effective publication: de la Haba *et al*., *Front Microbiol* 2023;14:1293707).
- *Halomonas lutescens* Wang *et al*. 2016 was reclassified as *Vreelandella lutescens* comb. nov. (Validation List 216; effective publication: de la Haba *et al*., *Front Microbiol* 2023;14:1293707).
- *Halomonas malpeensis* Kämpfer *et al*. 2018 was reclassified as *Vreelandella malpeensis* comb. nov. (Validation List 216; effective publication: de la Haba *et al*., *Front Microbiol* 2023;14:1293707).
- *Halomonas maris* Qiu *et al*. 2024 was reclassified as *Vreelandella maris* comb. nov. (Validation List 216; effective publication: de la Haba *et al*., *Front Microbiol* 2023;14:1293707).
- *Halomonas meridiana* James *et al*. 1990 was proposed as a heterotypic synonym of *Halomonas aquamarina* (ZoBell and Upham 1944) Dobson and Franzmann 1996 (de la Haba *et al., Front Microbiol* 2023;14:1293707).
- *Halomonas montanilacus* Lu *et al*. 2020 was reclassified as *Billgrantia montanilacus* comb. nov. (Validation List 216; effective publication: de la Haba *et al*., *Front Microbiol* 2023;14:1293707).
- *Halomonas muralis* Heyrman *et al*. 2002 was reclassified as *Modicisalibacter muralis* comb. nov. (Validation List 216; effective publication: de la Haba *et al*., *Front Microbiol* 2023;14:1293707).
- *Halomonas nanhaiensis* Long *et al*. 2013 was reclassified as *Vreelandella nanhaiensis* comb. nov. (Validation List 216; effective publication: de la Haba *et al*., *Front Microbiol* 2023;14:1293707).
- *Halomonas neptunia* Kaye *et al*. 2004 was reclassified as *Vreelandella neptunia* comb. nov. (Validation List 216; effective publication: de la Haba *et al*., *Front Microbiol* 2023;14:1293707).
- *Halomonas nigrificans* Oguntoyinbo *et al*. 2018 was reclassified as *Vreelandella nigrificans* comb. nov. (Validation List 216; effective publication: de la Haba *et al*., *Front Microbiol* 2023;14:1293707).
- *Halomonas niordana* Diéguez *et al*. 2020 was reclassified as *Onishia niordana* comb. nov. (Validation List 216; effective publication: de la Haba *et al*., *Front Microbiol* 2023;14:1293707).
- *Halomonas olivaria* Amouric *et al*. 2014 was reclassified as *Vreelandella olivaria* comb. nov. (Validation List 216; effective publication: de la Haba *et al*., *Front Microbiol* 2023;14:1293707).
- *Halomonas pacifica* (Baumann *et al*. 1972) Dobson and Franzmann 1996 was reclassified as *Bisbaumannia pacifica* comb. nov. (Validation List 216; effective publication: de la Haba *et al*., *Front Microbiol* 2023;14:1293707).
- *Halomonas pantelleriensis* Romano *et al*. 1997 was reclassified as *Franzmannia pantelleriensis* comb. nov. (Validation List 216; effective publication: de la Haba *et al*., *Front Microbiol* 2023;14:1293707).
- *Halomonas pellis* Li *et al*. 2020 was reclassified as *Billgrantia pellis* comb. nov. (Validation List 216; effective publication: de la Haba *et al*., *Front Microbiol* 2023;14:1293707).
- *Halomonas piezotolerans* Yan *et al*. 2020 was reclassified as *Vreelandella piezotolerans* comb. nov. (Validation List 216; effective publication: de la Haba *et al*., *Front Microbiol* 2023;14:1293707).
- *Halomonas populi* Xu *et al*. 2022 was reclassified as *Vreelandella populi* comb. nov. (Validation List 216; effective publication: de la Haba *et al*., *Front Microbiol* 2023;14:1293707).
- *Halomonas profundi* Wang *et al*. 2022 was reclassified as *Vreelandella profundi* comb. nov. (Validation List 216; effective publication: de la Haba *et al*., *Front Microbiol* 2023;14:1293707).
- *Halomonas qiaohouensis* Wang *et al*. 2015 was reclassified as *Franzmannia qiaohouensis* comb. nov. (Validation List 216; effective publication: de la Haba *et al*., *Front Microbiol* 2023;14:1293707).
- *Halomonas qijiaojingensis* Chen *et al*. 2012 was reclassified as *Litchfieldella qijiaojingensis* comb. nov. (Validation List 216; effective publication: de la Haba *et al*., *Front Microbiol* 2023;14:1293707).
- *Halomonas radicis* Navarro-Torre *et al*. 2020 was reclassified as *Modicisalibacter radicis* comb. nov. (Validation List 216; effective publication: de la Haba *et al*., *Front Microbiol* 2023;14:1293707).
- *Halomonas rifensis* Amjres *et al*. 2011 was reclassified as *Litchfieldella rifensis* comb. nov. (Validation List 216; effective publication: de la Haba *et al*., *Front Microbiol* 2023;14:1293707).
- *Halomonas rituensis* Gao *et al*. 2020 was reclassified as *Vreelandella rituensis* comb. nov. (Validation List 216; effective publication: de la Haba *et al*., *Front Microbiol* 2023;14:1293707).
- *Halomonas salicampi* Lee *et al*. 2015 was reclassified as *Vreelandella salicampi* comb. nov. (Validation List 216; effective publication: de la Haba *et al*., *Front Microbiol* 2023;14:1293707).
- *Halomonas salina* (Valderrama *et al*. 1991) Dobson and Franzmann 1996 was proposed as a heterotypic synonym of *Halomonas halophila* (Quesada *et al*. 1984) Dobson and Franzmann 1996 (de la Haba *et al., Front Microbiol* 2023;14:1293707).
- *Halomonas saliphila* Gan *et al*. 2018 was reclassified as *Billgrantia saliphila* comb. nov. (Validation List 216; effective publication: de la Haba *et al*., *Front Microbiol* 2023;14:1293707).
- *Halomonas sedimenti* Qiu *et al*. 2024 was reclassified as *Vreelandella sedimenti* comb. nov. (Validation List 216; effective publication: de la Haba *et al*., *Front Microbiol* 2023;14:1293707).
- *Halomonas songnenensis* Jiang *et al*. 2014 was reclassified as *Vreelandella songnenensis* comb. nov. (Validation List 216; effective publication: de la Haba *et al*., *Front Microbiol* 2023;14:1293707).
- *Halomonas stevensii* Kim *et al*. 2010 was reclassified as *Vreelandella stevensii* comb. nov. (Validation List 216; effective publication: de la Haba *et al*., *Front Microbiol* 2023;14:1293707).
- *Halomonas subglaciescola* Franzmann *et al*. 1987 was reclassified as *Vreelandella subglaciescola* comb. nov. (Validation List 216; effective publication: de la Haba *et al*., *Front Microbiol* 2023;14:1293707).
- *Halomonas subterranea* Xu *et al*. 2007 was reclassified as *Vreelandella subterranea* comb. nov. (Validation List 216; effective publication: de la Haba *et al*., *Front Microbiol* 2023;14:1293707).
- *Halomonas sulfidaeris* Kaye *et al*. 2004 was reclassified as *Vreelandella sulfidaeris* comb. nov. (Validation List 216; effective publication: de la Haba *et al*., *Front Microbiol* 2023;14:1293707).
- *Halomonas taeanensis* Lee *et al*. 2005 was reclassified as *Onishia taeanensis* comb. nov. (Validation List 216; effective publication: de la Haba *et al*., *Front Microbiol* 2023;14:1293707).
- *Halomonas titanicae* Mann *et al*. 2010 was reclassified as *Vreelandella titanicae* comb. nov. (Validation List 216; effective publication: de la Haba *et al*., *Front Microbiol* 2023;14:1293707).
- *Halomonas utahensis* Sorokin and Tindall 2006 was reclassified as *Vreelandella halophila* comb. nov. (Validation List 223; effective publication: de la Haba *et al*., *Front Microbiol* 2023;14:1293707).
- *Halomonas venusta* (Baumann *et al*. 1972) Dobson and Franzmann 1996 was reclassified as *Vreelandella venusta* comb. nov. (Validation List 216; effective publication: de la Haba *et al*., *Front Microbiol* 2023;14:1293707).
- *Halomonas vilamensis* Menes *et al*. 2011 was reclassified as *Vreelandella vilamensis* comb. nov. (Validation List 216; effective publication: de la Haba *et al*., *Front Microbiol* 2023;14:1293707).
- *Halomonas xianhensis* Xhao *et al*. 2012 was reclassified as *Modicisalibacter xianhensis* comb. nov. (Validation List 216; effective publication: de la Haba *et al*., *Front Microbiol* 2023;14:1293707).
- *Halomonas xinjiangensis* Guan *et al*. 2010 was reclassified as *Litchfieldella xinjiangensis* comb. nov. (Validation List 216; effective publication: de la Haba *et al*., *Front Microbiol* 2023;14:1293707).
- *Halomonas zhanjiangensis* Chen *et al*. 2009 was reclassified as *Vreelandella zhanjiangensis* comb. nov. (Validation List 216; effective publication: de la Haba *et al*., *Front Microbiol* 2023;14:1293707).
- *Halomonas zhuhanensis* Gao *et al*. 2020 was reclassified as *Vreelandella zhuhanensis* comb. nov. (Validation List 216; effective publication: de la Haba *et al*., *Front Microbiol* 2023;14:1293707).
- *Halomonas zincidurans* Xu *et al*. 2013 was reclassified as *Modicisalibacter zincidurans* comb. nov. (Validation List 216; effective publication: de la Haba *et al*., *Front Microbiol* 2023;14:1293707).
- *Modicisalibacter* Ben Ali Gam *et al*. 2007 emend. de la Haba *et al*. 2023 (*Front Microbiol* 2023;14:1293707).

R.R. de la Haba commented on the differences between the latest classifications in the publications by de la Haba *et al*. 2023 (*Front Microbiol* 2023;14:1293707) and Flores-Felix *et al*. 2025 (*Mol Phylogenet Evol* 2025;206:108321), and how these differences are reflected in large nomenclature and sequence repositories, such as Genome Taxonomy Database (GTDB), LPSN and National Center for Biotechnology Informatiion (NCBI) GenBank, highlighting what they have in common, as well as discrepancies. As of 1 November 2025, including names published in the October 2025 issue of the International Journal of Systematic and Evolutionary Microbiology (IJSEM) and Validation List no. 225, the family *Halomonadaceae* contained 18 genera and 185 species (genus names and number of species with validly published names, not including synonyms). The numbers above are based on: *Halomonas* (type genus of the family, 68), *Aidingimonas* (2), *Billgrantia* (18), *Bisbaumannia* (1), *Carnimonas* (1), *Chromohalobacter* (8), *Cobetia* (3), *Franzmannia* (2), *Halotalea* (1), *Kushneria* (10), *Larsenimonas* (3), *Litchfieldella* (4), *Modicisalibacter* (7), *Onishia* (2), *Pistricoccus* (1), *Salinicola* (13), *Vreelandella* (40) and *Zymobacter* (1). However, the taxonomic affiliation of *Terasakiispira* and other genera such as *Marinospirillum*, *Oceanospirillum* and *Allopseudospirillum,* and their possible inclusion in the family, requires further investigation.

The following name of a new genus of *Halomonadaceae* was effectively published since the Alicante 2022 subcommittee meeting:

- ‘*Cernens*’ Botero *et al*. 2025 (*Syst Appl Microbiol* 2025;48;126648), with type species ‘*Cernens ardua*’.

## Minute 11: recommended minimal standards for the description of new taxa

A. Ventosa explained the importance of recommended minimal standards for the description of new taxa of *Halobacteria* and *Halomonadaceae*. The minimal standards document for the *Halobacteria* was recently updated (Cui *et al.*, Int J Syst Evol Microbiol 2024;74:006290). The current minimal standards document for the *Halomonadaceae *(Arahal *et al.*, Int J Syst Evol Microbiol 2007;57:2436-2446) is quite old, and updating this document in the near future is recommended.

## Minute 12: highlighting recent papers of special interest

The following recently published papers of importance to the work of the two subcommittees were highlighted:

- Gupta RS, Rudra B, Tony J, Bello S. Phylogenomic and comparative analyses on protein sequences from *Halobacteria* to identify taxon-specific molecular markers which demarcate different *Halobacteriaceae* and *Haloarculaceae* genera. *Int J Syst Evol Microbiol* 2025;75:006879.
- Yao W, Zhang W, He W, Xiao W, Chen Y *et al*. Lipidomic chemotaxonomy aligned with phylogeny of *Halobacteria. Front Microbiol* 2023;14:1297600.
- Pfeiffer F, Dyall-Smith M. Genome comparison reveals that *Halobacterium salinarum* 63‐R2 is the origin of the twin laboratory strains NRC‐1 and R1. *MicrobiologyOpen* 2023;12:e1365.
- de la Haba RR, Arahal DR, Sánchez-Porro C, Chuvochina M, Wittouck S *et al*. A long-awaited taxogenomic investigation of the family *Halomonadaceae. Front Microbiol* 2023;14:1293707.

## Minute 13: next meeting of the subcommittees

The next joint meeting of both subcommittees is scheduled in association with the Halophiles 2028 conference, to be held in Messina, Italy.

## Minute 14: any other business

There was no other business.

## Minute 15: adjournment

The meeting was adjourned at 16.30 on 24 November 2025.

## Abbreviations

BAM: Bacteriology and Applied Microbiology
ICSP: International Committee on Systematics of Prokaryotes
IUMS: International Union of Microbiological Societies
LPSN: List of Prokaryote Names with Standing in Nomenclature

